# Exosomal ANXA2 derived from ovarian cancer cells regulates epithelial‐mesenchymal plasticity of human peritoneal mesothelial cells

**DOI:** 10.1111/jcmm.16983

**Published:** 2021-11-01

**Authors:** Lingling Gao, Xin Nie, Rui Gou, Yuexin Hu, Hui Dong, Xiao Li, Bei Lin

**Affiliations:** ^1^ Department of Obstetrics and Gynecology Shengjing Hospital of China Medical University Shenyang China; ^2^ Key Laboratory of Maternal‐Fetal Medicine of Liaoning Province Key Laboratory of Obstetrics and Gynecology of Higher Education of Liaoning Province Shenyang China

**Keywords:** ANXA2, epithelial‐mesenchymal plasticity, exosome, Implantation and metastasis, ovarian cancer

## Abstract

Ovarian cancer, one of the malignant gynaecological tumours with the highest mortality rate among female reproductive system, is prone to metastasis, recurrence and chemotherapy resistance, causing a poor prognosis. Exosomes can regulate the epithelial‐mesenchymal plasticity of tumour cells, remodel surrounding tumour microenvironment, and affect tumour cell proliferation, invasion and metastasis. However, the function and mechanism of exosomes in the intraperitoneal implantation of ovarian cancer remain unclear. In this study, exosomal annexin A2 (ANXA2) derived from ovarian cancer cells was co‐cultured with human peritoneal mesothelial (HMrSV5) cells; functional experiments were conducted to explore the effects of exosomal ANXA2 on the biological behaviour of HMrSV5 and the related mechanisms. This study showed that ANXA2 in ovarian cancer cells can be transferred to HMrSV5 cells through exosomes, exosomal ANXA2 can not only promote the migration, invasion and apoptosis of HMrSV5 cells, but also regulates morphological changes and fibrosis of HMrSV5 cells. Furthermore, ANXA2 promotes the mesothelial‐mesenchymal transition (MMT) and degradation of the extracellular matrix of HMrSV5 cells through PI3K/AKT/mTOR pathway, finally affects pre‐metastasis microenvironment of ovarian cancer, which provides a new theoretical basis for the mechanism of intraperitoneal implantation and metastasis of ovarian cancer.

## 1 INTRODUCTION

Ovarian cancer is regarded as one of most common gynaecological tumours with the highest mortality rate among gynaecological maligancy.^1^ Extensive pelvic and abdominal metastasis belong to the highly lethal cause in a majority of patients diagnosed at advanced stages (stages III–IV).^2^, ^3^ The process of ovarian cancer peritoneal metastasis was involved in multi‐steps including local infiltration, abdominal dissemination, distant invasion, anchoring and adhesion to peritoneum, and synergy with the tumour microenvironment.^4^, ^5^ In above process, peritoneal mesothelial cells, as the main components of the human peritoneum,^6^, ^7^ could create a suitable environment for tumour intraperitoneal implantation and metastasis through the change of biological properties.^8^, ^9^ Accumulating studies have suggested that various tumour cells could modulate the characteristics of peritoneal mesothelial cells by secreting various cytokines and bioactive substances, thus leading to intraperitoneal implantation.^10^, ^11^, ^12^ Therefore, a better understanding of the underlying molecular mechanism of malignant progression and peritoneal metastasis of ovarian cancer are essential to improve the survival and prognosis of patients and explore new therapeutic targets with ovarian cancer.

Exosomes, with a lipid bilayer membrane, were regarded as microvesicles with a diameter of about 30–150 nm, and widely present in a variety of body fluids, such as blood, urine, ascites, milk, bile and saliva.^13^ Exosomes could carry various biologically active substances, including nucleic acids (e.g., mRNA, miRNA, lncRNA, circRNA and DNA), lipids and proteins,^14^, ^15^, ^16^ which were involved in numbers of physiological activities and pathological processes, such as signal communication, molecular transport, immune response and antigen presentation.^17^ In recent years, many studies have shown that exosomes participated in the regulation of proliferation, invasion, metastasis and epithelial‐mesenchymal transition (MMT) of tumour cells, remodelling tumour environment, chemotherapy resistance and angiogenesis,^18^, ^19^ including gastric cancer, lung cancer and ovarian cancer. Especially, exosomes derived from cancer cells have potential abilities to target their parent cancer cells through delivering anti‐tumour drugs.^20^ Moreover, studies have shown that exosomal non‐coding RNA were frequently regarded as potential prognostic biomarkers, including miRNA‐200 family, miRNA‐141 and miRNA‐205,^21^, ^22^ while investigations of the function and mechanism of proteins in exosomes have been limited.

Annexin A2 (ANXA2), as calcium‐dependent membrane phospholipid‐binding protein, participates in cell migration, inflammation, fibrinolysis, exocytosis and endocytosis. As an extracellular fibrinolytic receptor, ANXA2 can not only promote proteolysis, neovascularization, invasion and metastasis of tumour cells, but remodel the extracellular matrix23‐24.^23^, ^24^ Our previous research has confirmed that ANXA2 was significantly associated with FIGO stages, pathological grade, lymph node metastasis and poor prognosis of ovarian cancer.^25^, ^26^ What is more, overexpression of ANXA2 promoted the invasion and metastasis of ovarian cancer cells and the tumorigenesis ability of transplanted tumours in nude mice. In recent years, proteomic studies also documented that ANXA2 was regarded as an important functional protein in exosomes secreted by bladder, colorectal and ovarian cancer cells.^27^ ANXA2 can regulate the formation and transport of extracellular vesicles,^28^ and miRNA can regulate the level of ANXA2 in exosome.^29^ However, the function and mechanism of exosomal ANXA2 on invasion, metastasis and abdominal implantation of ovarian cancer remain unclear.

In this study, we focused on the effect and potential mechanism of exosomal ANXA2 derived from ovarian cancer cells on peritoneal implantation and metastasis of tumours. The results revealed that exosomal ANXA2 derived from ovarian cancer could confer tumour characteristics to HMrSV5 cells and regulate MMT and extracellular matrix degradation of HMrSV5 cells, finally forming pre‐metastasis microenvironment suitable for intraperitoneal implantation and metastasis of ovarian cancer, which not only provide new insights into the molecular mechanism of intraperitoneal implantation and metastasis of ovarian cancer, but may also guide the development of therapeutic treatments to delay or inhibit tumour metastasis.

## 2 MATERIALS AND METHODS

### 2.1 Cell culture

Ovarian cancer cell lines, CaoV3, OVCAR3 and SKOV3, and normal peritoneal mesothelial cells (HMrSV5) were cultured in RPMI 1640 medium (BI, USA) supplemented with 10% foetal bovine serum (FBS) (BI, USA); ovarian cancer cell line ES‐2 were cultured in McCoy's 5A medium (BI, USA) supplemented with 10% FBS and incubated at 37°C and 5% CO_2_. Follow‐up experiments were carried out under the normal conditions.

### 2.2 Cell transfection and cell groups

For stable transfection of OVCAR3 and ES‐2 ovarian cancer cells and HMrSV5 cells, lentivirus‐mediated vector of *ANXA2* to inhibit or augment the expression of the *ANXA2* gene was designed and synthesized by Hanbio, and the procedure of transfections was conducted according to the manufacturer's instructions. Briefly, the volume of lentivirus to be transfected was calculated according to the infection values of different cells, the number of cells at the time of transfection and the lentivirus titre. 2 μg of polybrene(200 μg/ml)was added to the culture medium. After transfection for 24 or 48 h, stable transfected cells were further screened by puromycin (2 μg/ml) and verified by Western blot and real‐time PCR. The transfection groups were as follows: OVCAR3 (blank group), OVCAR3‐NC (negative control group), OVCAR3‐shANXA2 (ANXA2 downregulated group), OVCAR3‐ON (negative control group) and OVCAR3‐ANXA2‐H (ANXA2 upregulated group); ES‐2 (blank group), ES‐2‐NC (negative control group), ES‐2‐shANXA2 (ANXA2 downregulated group), ES‐2‐ON (negative control group) and ES‐2‐ANXA2‐H (ANXA2 upregulated group); HMrSV5 (blank group), HMrSV5‐NC (negative control group), HMrSV5‐shANXA2 (ANXA2 downregulated group), HMrSV5‐ON (negative control group) and HMrSV5‐ANXA2‐H (ANXA2 upregulated group).

### 2.3 Exosome extraction

Ovarian cancer cells were cultured to 70%–80% confluence (10‐cm dish) with complete culture medium. Cells were washed with PBS three times after the supernatant was removed, and then routinely cultured in fresh serum‐free medium for 48 h. The culture supernatant was collected and centrifuged at 300 *g* for 10 min at 4°C to remove dead cells and fragments in the culture supernatant. Then, the culture supernatant was centrifuged at 2,000 *g* for 10 min at 4°C to remove biopolymers and apoptotic bodies. A Millipore aseptic filter (pore size: 0.22 μm; Millipore) was used to remove large vesicles and particles as well as bacteria. Then, the filtered supernatant was centrifugated at 10,000 *g* for 30 min at 4°C to remove cell fragments, large vesicles and impurities. The precipitation at the bottom of the tube was discarded, and the supernatant was centrifuged at 100,000 *g* for 90 min at 4°C. The supernatant was removed, and the exosome precipitation was re‐suspended in 50–100 μl PBS and stored at −80°C. The groups were as follows: OVCAR3‐exo and ES‐2‐exo are exosomes derived from cells in the blank group; OVCAR3‐NC‐exo and ES‐2‐NC‐exo are exosomes derived from cells in the negative control groups; OVCAR3‐shANXA2‐exo and ES‐2‐shANXA2‐exo are exosomes derived from cells in the ANXA2 downregulated expression groups; OVCAR3‐ON‐exo and ES‐2‐ON‐exo are exosomes derived from cells in the negative control groups; OVCAR3‐ANXA2‐H‐exo and ES‐2‐ANXA2‐H‐exo are exosomes derived from the ANXA2 upregulated expression groups.

### 2.4 Transmission electron microscope (TEM)

The morphology and structure of the exosomes in the supernatant of each cell culture were identified by TEM. The exosomes were re‐suspended in 100 μl PBS and stored at 4°C. Approximately 10–20 μl of exosome suspension was added to a carbon‐coated copper mesh matched by the electron microscope. The copper mesh was exposed to room temperature for 30 min to precipitate the exosomes. Filter paper was used to absorb the liquid from the side of the filter. The exosomes were then negatively stained with 3% phosphotungstic acid dye solution (20–50 μl) for 20 min. After the filter paper was dried, the exosomes were washed with PBS three times. After drying at room temperature, the exosomes were observed and photographed under TEM.

### 2.5 Nanoparticle Tracking Analysis (NTA)

The extracted exosome precipitate was re‐suspended with 100 μl PBS and stored at 4°C. The exosome sample was diluted with PBS and fully mixed. The mixed exosome sample (300 μl) was detected by Electrophoresis & Brownian Motion Video Analysis Laser Scattering Microscopy, the particle size and purity of the exosomes were analysed by Zetaview visual nanoparticle tracer.

### 2.6 Exosome fluorescence labelling uptake experiment

To determine whether the exosomes from ovarian cancer cells can be taken up by HMrSV5, 10 mg Dil dye (Solarbio) was adjusted to a concentration of 2 mg/ml with 5 ml dimethyl sulfoxide (DMSO). The purified exosomes were suspended in 200 μl PBS, 2 mg/ml Dil dye was added, and the working concentration was adjusted to 15 μM. The mixture was incubated at room temperature for 30 min and then centrifuged at 100,000 *g* for 90 min to remove unbound dye, washed with PBS for three times. The supernatant was discarded, and Dil‐labelled exosomes were re‐suspended in 200 μl PBS, and then co‐incubated with HMrSV5 for 6 h or 12 h, respectively. HMrSV5 cells co‐cultured with Dil‐labelled exosomes were fixed with 4% paraformaldehyde (Solarbio) for 20 min at room temperature and washed with PBS three times. Then, 500 μl 4′,6‐diamidino‐2‐phenylindole dihydrochloride (DAPI) solution (1:1000 dilution with PBS) was incubated for 5 min and then washed with PBS for three times. The condition of exosomes taken up by cells was observed by laser confocal microscope.

### 2.7 Western blot

Cell precipitation was lysed with RIPA lysis buffer supplemented with phenylmethylsulfonyl fluoride (PMSF) at 4°C for 30 min. After centrifugation at 4°C and 13800 *g* for 20 min, the concentration of protein in the supernatant was detected by a bicinchoninic acid assay (BCA; Thermo Fisher Scientific). Proteins were separated by 10% SDS‐PAGE and then transferred onto PVDF membrane with 0.22‐μm pore size (Millipore). After blocking with 5% skim milk or bovine serum albumin for 2 h, the membranes were incubated overnight at 4°C with primary antibodies as follows: ANXA2 (1:5000; Proteintech, Rosemont), β‐actin (1:2000; Proteintech), CD63 (1:500; Abcam), tumour susceptibility gene 101 (TSG101) (1:500; Cell Signaling Technology [CST]), ALG‐2‐interacting protein X (Alix) (1:500; CST), calnexin (1:500; Proteintech), E‐cadherin (1:2000; Proteintech), N‐cadherin (1:1000; Proteintech), vimentin (1:4000; Proteintech), MMP2 (1:1000; Proteintech), MMP9 (1:1000; Proteintech), fibronectin (1:1000; Proteintech), α‐SMA (1:1000; Proteintech), Bcl2 (1:1000; Proteintech), BAX (1:1000; Proteintech), proliferating cell nuclear antigen (PCNA) (1:1000; Proteintech), PI3K (1:500; Proteintech), *p*‐PI3K (1:500; CST), AKT (1:1000; CST), *p*‐AKT (1:1000; CST), mTOR (1:1000; CST) and *p*‐mTOR (1:1000; CST). The PVDF membranes were washed three times with 1 × tris‐buffered saline and Tween 20 for 10 min and then incubated with anti‐mouse/rabbit IgG antibody (1:2000) for 2 h at room temperature. After washing with 1 × tris‐buffered saline (TBS) and Tween 20 for 10 min, the protein bands were visualized with ECL Luminescent Reagent (Millipore). The experiment was repeated three times.

### 2.8 Real‐time PCR

Total RNA was extracted by Trizol Reagent (Invitrogen) and quantified, reverse transcription was conducted according to the instructions of the PrimeScript RT reagent kit with gDNA Eraser (Takara Bio). The following procedure was implemented with SYBR Premix Ex Taq II kit (Takara Bio). The PCR reaction was carried out on a fluorescence quantitative PCR instrument (7500 Real‐Time PCR Detection System; Applied Biosystems). The data were statistically analysed with ACTB as the internal control and 2^−ΔΔCt^ as the relative expression. The sequence of primers used was as follows: ACTB forward primer: 5′‐CCTGGCACCCAGCACAAT‐3′, ACTB reverse primer: 3′‐GGGCCGGACTCGTCATAC‐5′; ANXA2 forward primer 5′‐TCTACTGTTCACGAAATCCTGTG‐3′, ANXA2 reverse primer: 5′‐ AGTATAGGCTTTGACAGACCCAT‐3′. The experiment was repeated three times.

### 2.9 Cell migration ability detected by scratch test

The cells in logarithmic phase were routinely digested with trypsin and seeded in a 6‐well culture plate. When the cells reached 90% confluence, the cell layer was gently scratched using a 100 μl micropipette tip, washed with PBS to remove floated cells, and then cultured in serum‐free medium for 48 h at 37°C. The wound‐healing ability of the cells was observed and photographed under a microscope at 0 and 48 h. This experiment was repeated three times.

### 2.10 Cell invasion ability detected by Transwell assay

Invasion ability was assessed by Transwell assay (Corning Coster) in a 24‐well culture plate. Matrigel (BD Corporation) was diluted with serum‐free medium or PBS buffer (serum‐free medium or PBS: Matrigel = 8:1). The cells in the exponential growth phase were digested routinely, and the cell concentration was adjusted to about 10^5^ cells/ml. Approximately 200 μl single‐cell suspension (about 2 × 10^4^ cells) was added to upper layer of the Transwell chamber, while 500 μl medium supplemented with 10% FBS was added to the lower chamber. After incubation at 37°C for 48 h, the cells in the Transwell chamber were fixed with 4% paraformaldehyde for 20 min and then stained with crystal violet for 30 min. A cotton swab was used to remove the Matrigel and cells from the upper surface of the chamber. The stained cells were observed and counted under a microscope. The experiments were repeated three times.

### 2.11 Cell apoptosis detected by flow cytometry

Cells in the exponential growth phase were digested with trypsin without EDTA and neutralized in complete medium. The single‐cell suspension was collected and centrifuged at 978 *g* for 5 min, and then washed with PBS for three times. The cells were stained with 500 μl binding buffer and gently mixed, after which 5 μl propidium iodide (PI) and 5 μl antigen‐presenting cell (APC)‐labelled Annexin V reagent (Annexin V‐APC/PI Apoptosis Detection Kit; Beyotime) was added and mixed thoroughly. The cells were then incubated for 15 min at room temperature. Apoptosis was detected by flow cytometry within 1 h. The above experiments were repeated three times.

### 2.12 Cell proliferation ability detected by CCK8

Cells in the logarithmic growth phase were digested with trypsin and formed a single‐cell suspension. A total of 2000 cells/200 μl were seeded in each well of a 96‐well plate. The exosomes derived from ovarian cancer cells in each group were added to the culture medium. The 96‐well plate was cultured in cell incubator at 37°C for 4 h, with 0 h marked as the time immediately after adherence. A volume of 10 μl CCK8 regent (Bimake) solution was added to each well, and the cells were cultured for 4 h at 37°C. The absorbance of cells at optical density 450 nm was measured after 24, 48, 72 and 96 h, respectively. The above experiments were repeated three times.

### 2.13 Cell cytoskeleton detected by Phalloidin staining

The cells in the exponential growth phase are routinely digested and centrifuged. When the cell density grows to 40%–50%, the culture medium is discarded and washed with PBS for three times. After fixed with 4% paraformaldehyde for 20 min and washed with PBS for three times. Cells were incubated with Triton X‐100 (Beyotime) for 10 min. Subsequently, the cells were incubated with the Actin‐Tracker Green‐488 (green fluorescent probe) (Beyotime) diluted in 1% BSA with PBS (1:100) for 1 h at room temperature. Next, the nuclei were stained by DAPI diluted with PBS (1:1000) for 10 min at room temperature. Cell cytoskeleton was observed under a laser confocal microscope.

### 2.14 Statistical analysis

All data were analysed by IBM SPSS Statistics V21.0 software (IBM Corp.) and expressed as mean ± standard deviation (variance ± SD). Comparisons between two groups were analysed by Student's *t*‐test. Single analysis of variance (ANOVA) was used to analyse comparisons among more than two groups. Bilateral *p*‐values < 0.05 were considered statistically significant.

## 3 RESULTS

### 3.1 Exosomal ANXA2 derived from OVCAR3 and ES‐2 ovarian cancer cells

**FIGURE 1:**
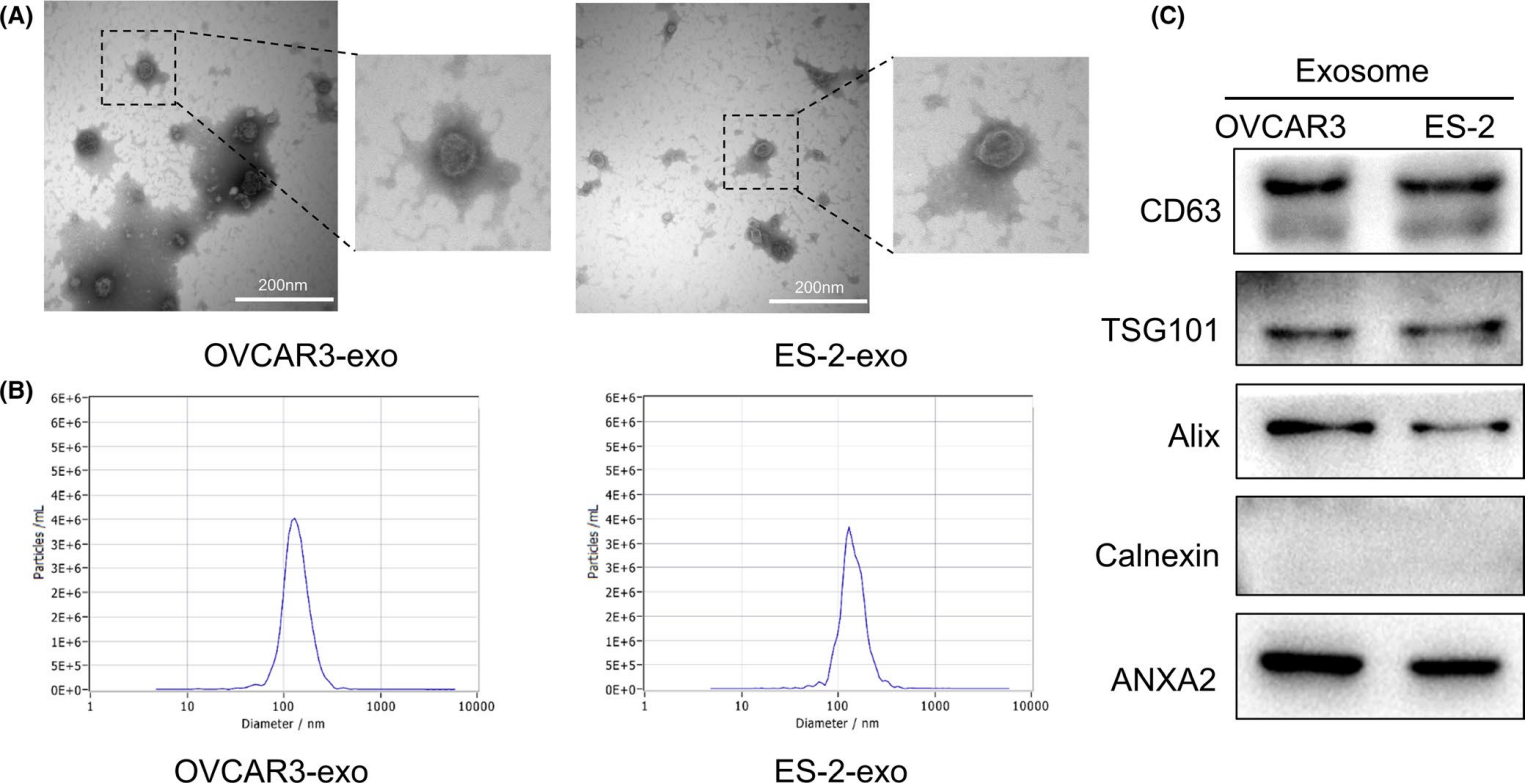
Exosomal ANXA2 derived from OVCAR3 and ES‐2 ovarian cancer cells. (A) Typical morphology of exosomes derived from OVCAR3 and ES‐2 cells observed by transmission electron microscope (TEM). (B) Particle size and purity of the exosomes detected by Nanoparticle Tracking Analysis (NTA). (C) Expression of exosome molecular marker proteins CD63, tumour susceptibility gene 101(TSG101), ALG‐2‐interacting protein X (Alix) and calnexin in the exosomes detected by Western blot. (D) Expression of ANXA2 in the exosomes derived from OVCAR3 and ES‐2 cells detected by Western blot

Exosomes derived from ovarian cancer cells were extracted by ultracentrifugation. The morphology of purified exosomes was observed by transmission electron microscope (TEM), which showed round or oval microvesicles with a diameter of about 30–150 nm. The microvesicles were present as tea‐like or concave hemispherical, consistent with the typical structural characteristics of exosome (Figure 1A, Figure S1). The particle size and purity of the exosomes were further analysed by nanoparticle tracking analysis (NTA) (Figure 1B). We detected the positive expression of molecular markers CD63 protein, tumour susceptibility gene 101 (TG101), and ALG‐2‐interacting protein X (Alix), and negative expression of the endoplasmic reticulum protein calnexin with Western blot. We further confirmed the expression of ANXA2 in exosomes derived from OVCAR3 and ES‐2 ovarian cancer cells with Western blot (Figure 1C).

### 3.2 Exosomal ANXA2 level and expression of ANXA2 in host cells

**FIGURE 2:**
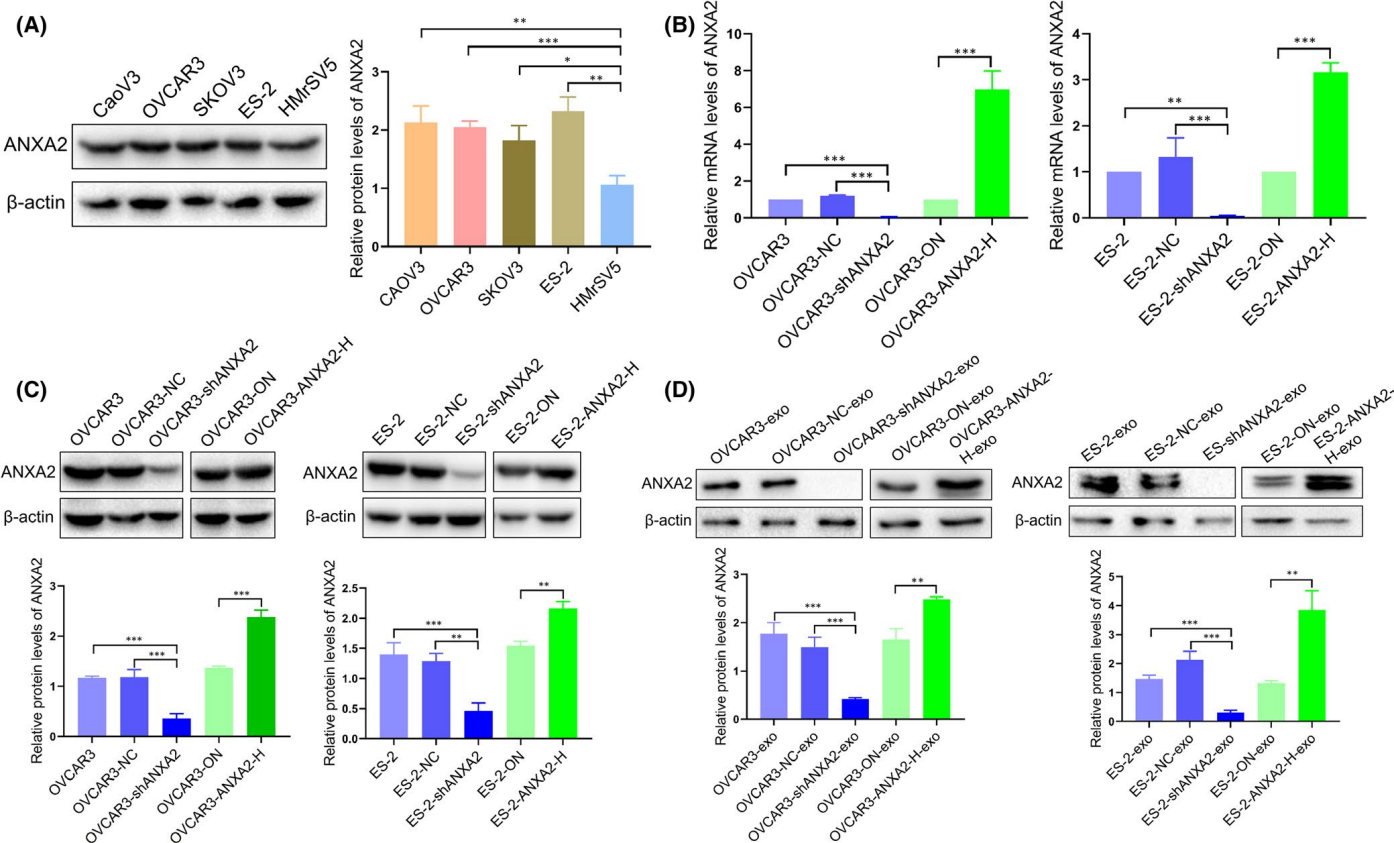
Exosomal ANXA2 level and expression of ANXA2 in host cells. (A) Expression of ANXA2 in CaoV3, OVCAR3, SKOV3 and ES‐2 ovarian cancer cells and human peritoneal mesothelial cells (HMrSV5) detected by Western blot. (B) mRNA levels of ANXA2 in OVCAR3 and ES‐2 cells with up‐ and downregulated ANXA2 detected by real‐time PCR. (C) Protein levels of ANXA2 in OVCAR3 and ES‐2 cells with up‐ and downregulated ANXA2 detected by Western blot. (D) Changes in exosomal ANXA2 derived from ovarian cancer cells with up‐ and downregulated ANXA2 detected by Western blot

We firstly detected the expression level of ANXA2 in CaoV3, OVCAR3, SKOV3 and ES‐2 ovarian cancer cells and HMrSV5 cells by Western blot. The expression of ANXA2 in ovarian cancer cells was significantly higher than that in HMrSV5 cells (*p* < 0.05; Figure 2A). OVCAR3 and ES‐2 ovarian cancer cells were further chosen to construct stable high and low expression of ANXA2 by lentiviral transfection. Western blot and real‐time PCR showed that the protein and mRNA levels of ANXA2 in the OVCAR3‐shANXA2 and ES‐2‐shANXA2 groups were significantly lower than those in the OVCAR3, OVCAR3‐NC, ES‐2 or ES‐2‐NC (all *p *< 0.05; Figure 2B,C). The protein and mRNA levels of ANXA2 in the OVCAR3‐ANXA2‐H and ES‐2‐ANXA2‐H groups were significantly higher than those in the OVCAR3‐ON or ES‐2‐ON (all *p *< 0.05; Figure 2B,C).

Ultracentrifugation was further utilized to extract exosomes derived from ovarian cancer cells with up‐ or downregulated ANXA2 expression. The level of exosomal ANXA2 protein was verified by Western blot. Exosomal ANXA2 protein in OVCAR3‐shANXA2‐exo group and ES‐2‐shANXA2‐exo group was significantly lower than those in OVCAR3‐exo group, OVCAR3‐NC‐exo, ES‐2‐exo group and ES‐2‐NC‐exo group (all *p *< 0.05; Figure 2D). Exosomal ANXA2 protein derived from the OVCAR3‐ANXA2‐H‐exo group and ES‐2‐ANXA2‐H‐exo group were significantly higher than those in OVCAR3‐ON‐exo group and ES‐2‐ON‐exo group (*p *< 0.05; Figure 2D).

### 3.3 Expression of ANXA2 and migration and invasion abilities of HMrSV5 cells co‐cultured with exosomes derived from OVCAR3 and ES‐2 cells

**FIGURE 3:**
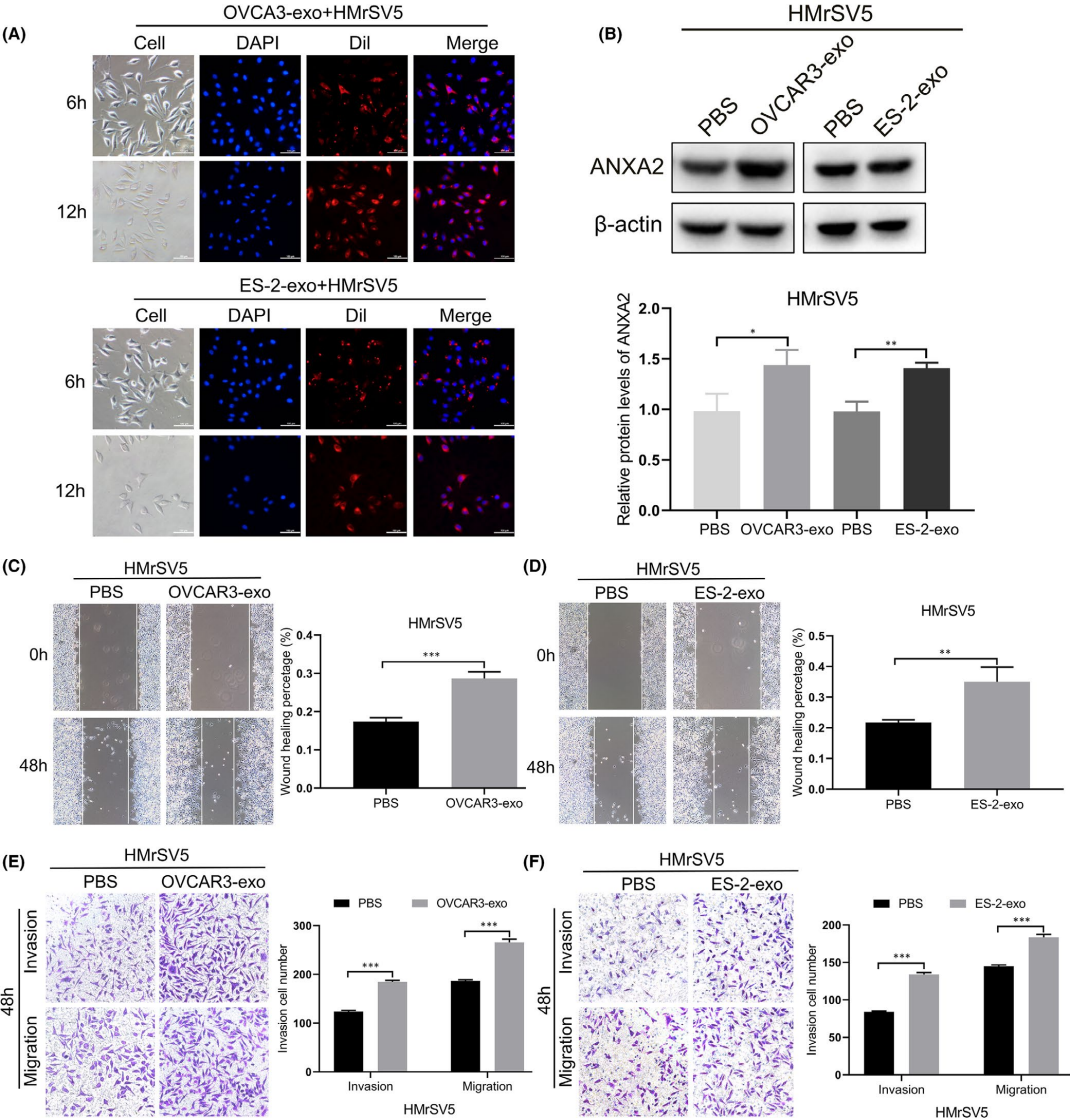
Expression of ANXA2 and migration and invasion abilities of HMrSV5 cells co‐cultured with OVCAR3‐exo and ES‐2‐exo. (A) Exosomes derived from OVCAR3 and ES‐2 cells (labelled with red Dil) taken up by human peritoneal mesothelial cells (HMrSV5), as seen under fluorescence microscope. Scale bar, 100 μm. (B) Expression of ANXA2 in HMrSV5 cells co‐cultured with OVCAR3‐exo and ES‐2‐exo detected by Western blot. (C) Migration ability of HMrSV5 cells co‐cultured with OVCAR3‐exo detected by scratch test. (D) Migration ability of HMrSV5 cells co‐cultured with ES‐2‐exo detected by scratch test. (E) Invasion ability of HMrSV5 cells co‐cultured with OVCAR3‐exo detected by Transwell assay. (F) Invasion ability of HMrSV5 cells co‐cultured with ES‐2‐exo detected by Transwell assay. *p < 0.05, **p < 0.01, ***p < 0.001

To investigate whether HMrSV5 cells can take up exosomes derived from ovarian cancer cells, Dil fluorescent‐labelled exosomes derived from ovarian cancer cells were co‐cultured with HMrSV5 cells for 6 and 12 h, respectively. The result showed that unevenly distributed red fluorescent granular was present in the cytoplasm of HMrSV5 cells for 6 h. After 12 h, the red fluorescence labelled exosomes gradually gathered around the nuclei of HMrSV5 cells, indicating that the uptake of exosomes by HMrSV5 cells increased gradually with the prolongation of time (Figure 3A).

We further explored the effect of OVCAR3‐exo and ES‐2‐exo on the expression of ANXA2 in HMrSV5 cells and their biological behaviours through co‐culturing OVCAR3‐exo or ES‐2‐exo with HMrSV5 cells. Western blot showed that the expression of ANXA2 in HMrSV5 cells co‐cultured with OVCAR3‐exo or ES‐2‐exo was upregulated compared with that in control group (Figure 3B). The levels of ANXA2 mRNA in HMrSV5 cells co‐cultured with OVCAR3‐exo or ES‐2‐exo did not change significantly compared with that in control group by real‐time PCR (*p *> 0.05, Figure S2A). Scratch test showed that migration capacities were significantly higher in HMrSV5 cells co‐cultured with OVCAR3‐exo or ES‐2‐exo than that in control group (both *p* < 0.05; Figure 3C,D). Transwell assay showed that the invasion capacities were significantly higher in HMrSV5 cells co‐cultured with OVCAR3‐exo or ES‐2‐exo than that in control group (both *p* < 0.05; Figure 3E,F).

### 3.4 Effect of GW4869 on the expression of ANXA2 and migration and invasion abilities of HMrSV5 cells co‐cultured with OVCAR3‐exo and ES‐2‐exo

**FIGURE 4:**
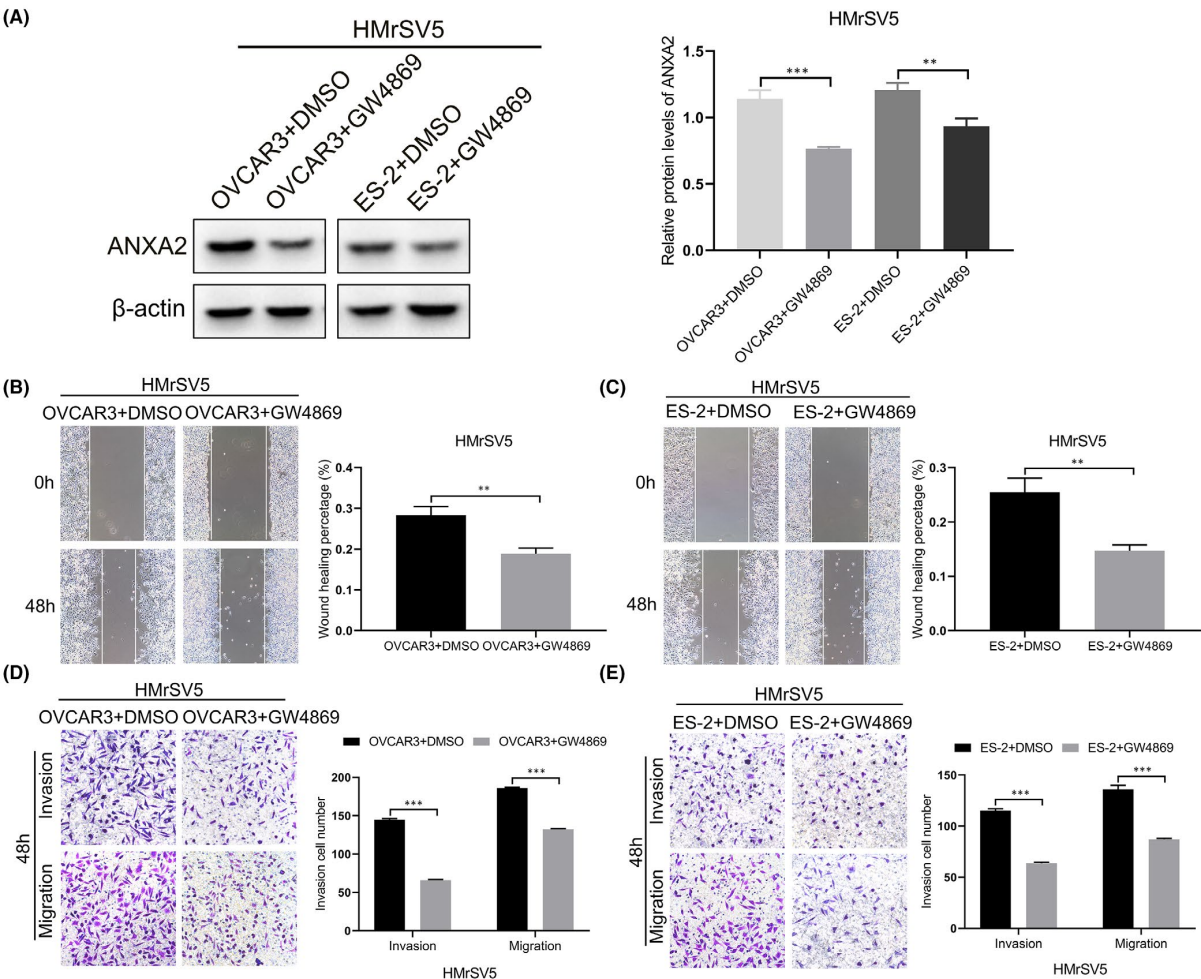
Effect of GW4869 on the expression of ANXA2 and migration and invasion abilities of HMrSV5 cells co‐cultured with exosomes derived from OVCAR3 and ES‐2 cells. (A) Expression of ANXA2 in HMrSV5 cells co‐cultured with exosomes derived from OVCAR3 and ES‐2 cells with GW4869 detected by Western blot. (B) Migration ability of HMrSV5 cells co‐cultured with exosomes derived from OVCAR3 cells with GW4869 detected by Scratch test. (C) Migration ability of HMrSV5 cells co‐cultured with exosomes derived from ES‐2 cells with GW4869 detected by Scratch test. (D) Invasion ability of HMrSV5 cells co‐cultured with exosomes derived from OVCAR3 cells with GW4869 detected by Transwell assay. (E) Invasion ability of HMrSV5 cells co‐cultured with exosomes derived from ES‐2 cells with GW4869 detected by Transwell assay. *p < 0.05, **p < 0.01, ***p < 0.001

We further added the exosome inhibitor GW4869 to the culture medium of ovarian cancer cells (OVCAR3+GW4869 and ES‐2+GW4869) to explore its effect on the expression of ANXA2 and biological behaviours of HMrSV5 cells. Western blot showed that, compared with those of cells in the control groups (OVCAR3+DMSO and ES‐2+DMSO), the expression levels of ANXA2 in HMrSV5 cells were downregulated in the OVCAR3+GW4869 and ES‐2+GW4869 groups after the addition of GW4869 (*p* < 0.05; Figure 4A). The levels of ANXA2 mRNA in HMrSV5 cells did not change significantly after addition of GW4869 in the culture medium by real‐time PCR (*p *> 0.05, Figure S2B). Scratch test showed that, compared with those in the control groups, migration abilities were inhibited in the OVCAR3+GW4869 and ES‐2+GW4869 groups after the addition of GW4869 (all *p* < 0.05; Figure 4B,C). Transwell assay showed that, compared with those in the control groups, invasion abilities were inhibited in the OVCAR3+GW4869 and ES‐2+GW4869 groups after the addition of GW4869 (all *p* < 0.05; Figure 4D,E).

### 3.5 Migration and invasion abilities of HMrSV5 cells co‐cultured with exosomal ANXA2 derived from ovarian cancer cells

**FIGURE 5:**
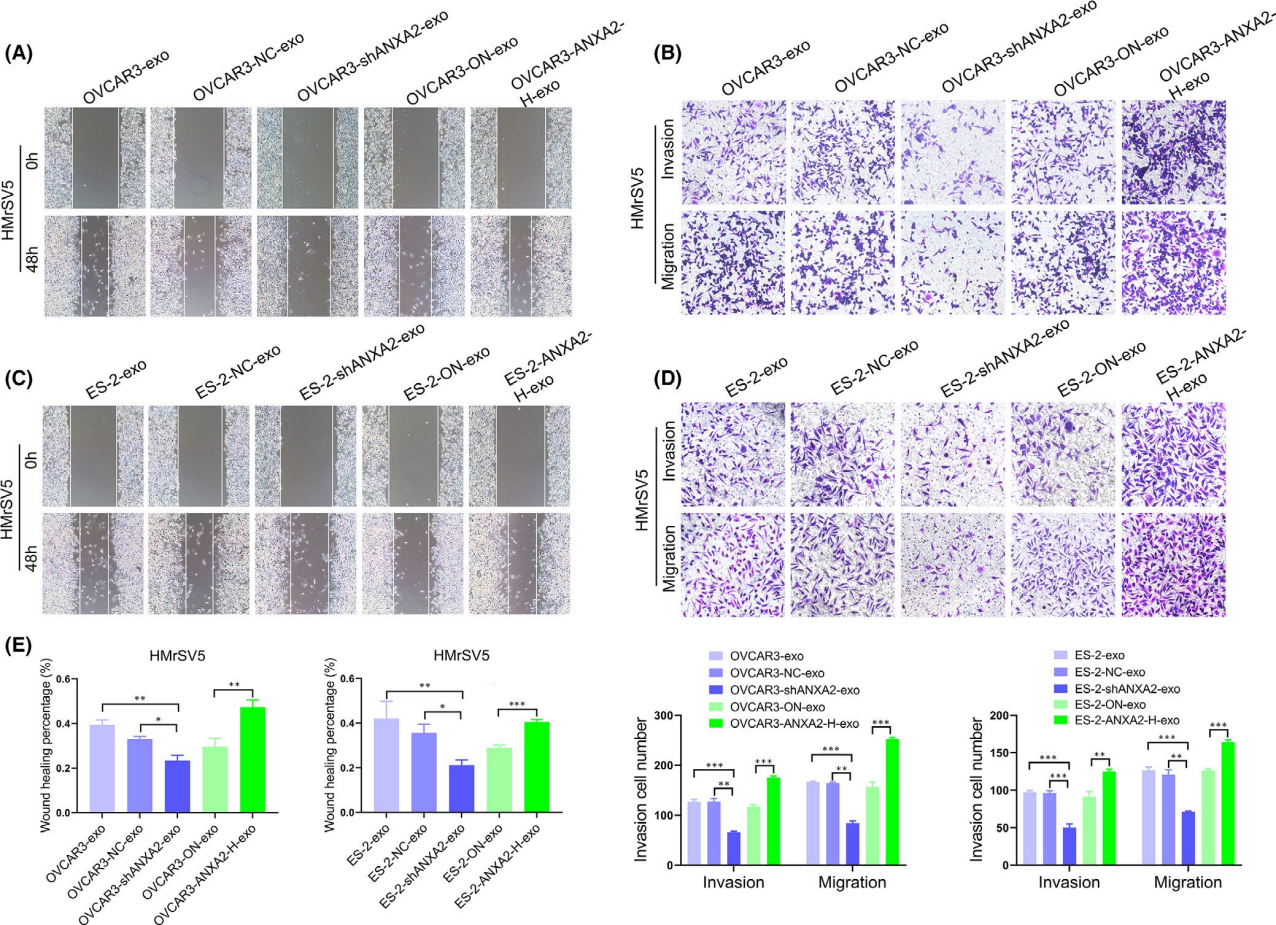
Migration and invasion abilities of HMrSV5 cells co‐cultured with exosomal ANXA2 derived from ovarian cancer cells. (A) Migration abilities of HMrSV5 cells co‐cultured with exosomal ANXA2 derived from OVCAR3 cells detected by Scratch test. (B) Migration abilities of HMrSV5 cells co‐cultured with exosomal ANXA2 derived from ES‐2 cells detected by Scratch test. (C) Invasion abilities of HMrSV5 cells co‐cultured with exosomal ANXA2 derived from OVCAR3 cells detected by Transwell assay. (D) Invasion abilities of HMrSV5 cells co‐cultured with exosomal ANXA2 derived from ES‐2 cells detected by Transwell assay. (E) The above data statistics charts were shown. *p < 0.05, **p < 0.01, ***p < 0.001

The influence of exosomal ANXA2 on biological characteristics of HMrSV5 cells was further detected through co‐cultured with exosomes derived from ovarian cancer cells with downregulated or upregulated ANXA2 protein. Scratch test showed that the migration abilities of HMrSV5 cells co‐cultured with OVCAR3‐shANXA2‐exo and ES‐2‐shANXA2‐exo were significantly decreased compared with those of cells co‐cultured with OVCAR3‐exo, OVCAR3‐NC‐exo, ES‐2‐exo and ES‐2‐NC‐exo (all *p *< 0.05; Figure 5A,B). Compared with the migration abilities of HMrSV5 cells co‐cultured with OVCAR3‐ON‐exo and ES‐2‐ON‐exo, those of HMrSV5 cells co‐cultured with OVCAR3‐ANXA2‐H‐exo and ES‐2‐ANXA2‐H‐exo were significantly enhanced (*p *< 0.05; Figure 5A,B,E). Transwell assay showed that the invasion abilities of HMrSV5 cells co‐cultured with OVCAR3‐shANXA2‐exo and ES‐2‐shANXA2‐exo were significantly decreased compared with those of HMrSV5 cells co‐cultured with OVCAR3‐exo, OVCAR3‐NC‐exo, ES‐2‐exo and ES‐2‐NC‐exo (all *p *< 0.05; Figure 5C,D). Compared with the invasion abilities of HMrSV5 cells co‐cultured with OVCAR3‐ON‐exo and ES‐2‐ON‐exo, those of HMrSV5 cells co‐cultured with OVCAR3‐ANXA2‐H‐exo and ES‐2‐ANXA2‐H‐exo were significantly enhanced (*p* < 0.05; Figure 5C–E).

### 3.6 Apoptosis and mesenchymal‐like changes in HMrSV5 cells co‐cultured with exosomal ANXA2 derived from ovarian cancer cells

**FIGURE 6:**
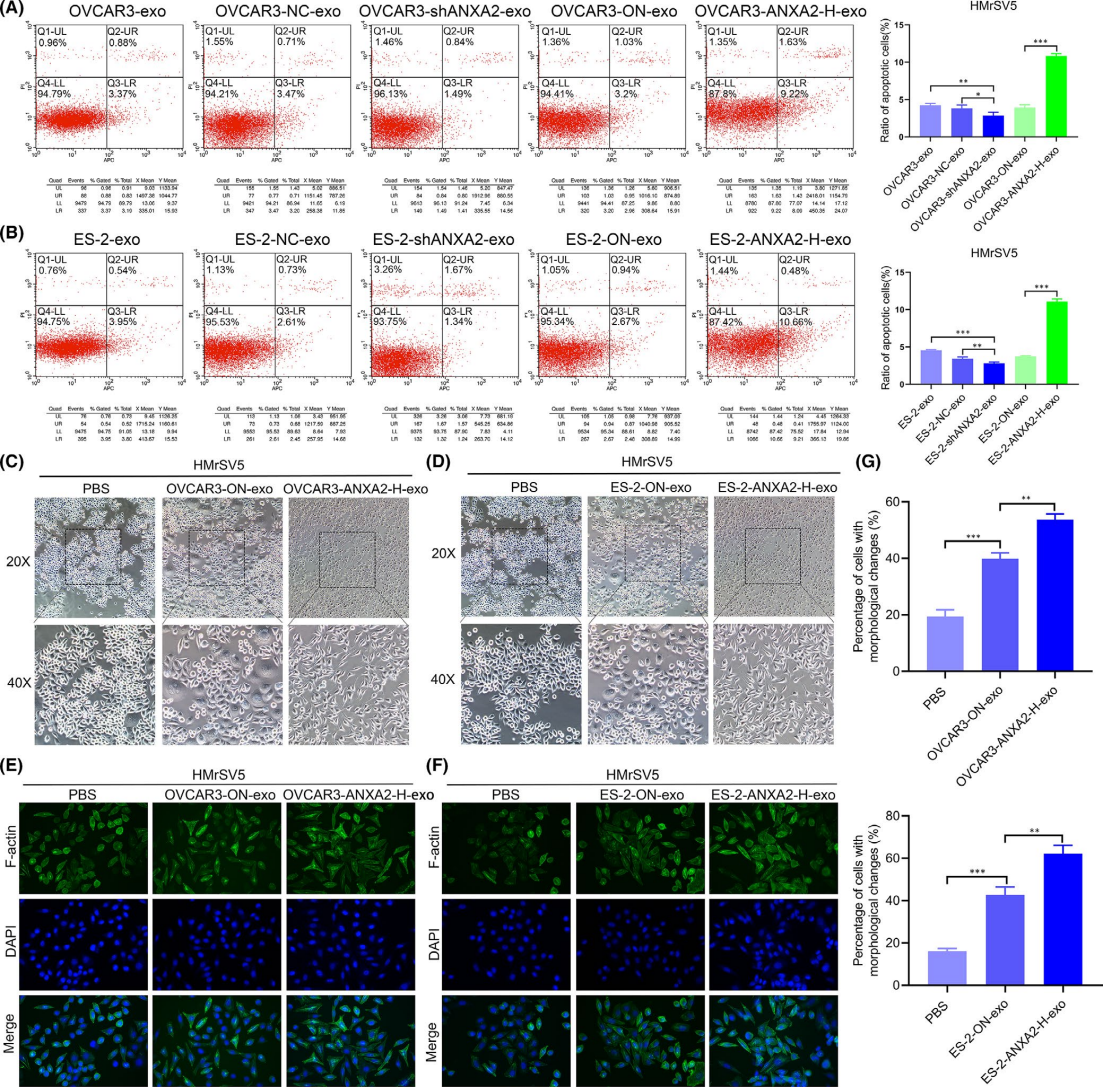
Apoptosis and mesenchymal‐like changes in HMrSV5 cells co‐cultured with exosomal ANXA2 derived from ovarian cancer cells. (A) Apoptotic abilities of HMrSV5 cells co‐cultured with exosomal ANXA2 derived from OVCAR3 cells detected by flow cytometry. (B) Apoptotic abilities of HMrSV5 cells co‐cultured with exosomal ANXA2 derived from ES‐2 cells detected by flow cytometry. Q1‐UL: necrotic cells; Q2‐UR: the late apoptotic cells; Q3‐LR: the early apoptotic cells; Q4‐LL: normal cells. (C) Morphology changes in HMrSV5 cells co‐cultured with exosomal ANXA2 derived from OVCAR3 cells observed under inverted phase contrast microscope. (D) Morphology changes in HMrSV5 cells co‐cultured with exosomal ANXA2 derived from ES‐2 cells observed under inverted phase contrast microscope. (E) Content and morphological changes in F‐actin in the cytoskeleton of HMrSV5 cells co‐cultured with exosomal ANXA2 derived from OVCAR3 cells detected by phalloidin staining. (F) Content and morphological changes in F‐actin in the cytoskeleton of HMrSV5 cells co‐cultured with exosomal ANXA2 derived from ES‐2 cells detected by phalloidin staining. (G) The statistical analysis of HMrSV5 cells with morphological changes after co‐cultured with exosomal ANXA2 derived from ovarian cancer cells. *p < 0.05, **p < 0.01, ***p < 0.001

Flow cytometry showed that the apoptotic ability of HMrSV5 cells co‐cultured with OVCAR3‐shANXA2‐exo and ES‐2‐shANXA2‐exo were significantly decreased compared with those of HMrSV5 cells co‐cultured with the OVCAR3‐exo, OVCAR3‐NC‐exo, ES‐2‐exo and ES‐2‐NC‐exo (all *p *< 0.05; Figure 6A,B). Compared with the apoptotic ability of HMrSV5 cells co‐cultured with OVCAR3‐ON‐exo and ES‐2‐ON‐exo, those of HMrSV5 cells co‐cultured with OVCAR3‐ANXA2‐H‐exo and ES‐2‐ANXA2‐H‐exo were significantly upregulated (*p *< 0.05; Figure 6A,B).

The effects of exosomal ANXA2 on morphology changes in HMrSV5 cells were observed under inverted phase contrast microscope. The results showed that, compared with that of cells in the PBS, OVCAR3‐ON‐exo and ES‐2‐ON‐exo groups, the morphology of HMrSV5 cells co‐cultured with OVCAR3‐ANXA2‐H‐exo or ES‐2‐ANXA2‐H‐exo changed from cobblestone‐like, regular and tightly arranged to long fusiform; intercellular spaces were enlarged and loosely arranged, and bare areas appeared (Figure 6C,D). Phalloidin staining showed that, compared with those of cells in the PBS, OVCAR3‐ON‐exo and ES‐2‐ON‐exo groups, the F‐actin content and the filopodia of HMrSV5 cells co‐cultured with OVCAR3‐ANXA2‐H‐exo and ES‐2‐ANXA2‐H‐exo were significantly increased, and the cytoskeleton structure changed to mesenchymal‐like morphology (Figure 6E–G).

### 3.7 Effect of ANXA2 on migration, invasion, mesothelial‐mesenchymal transition (MMT) and fibrosis of HMrSV5 cells

**FIGURE 7:**
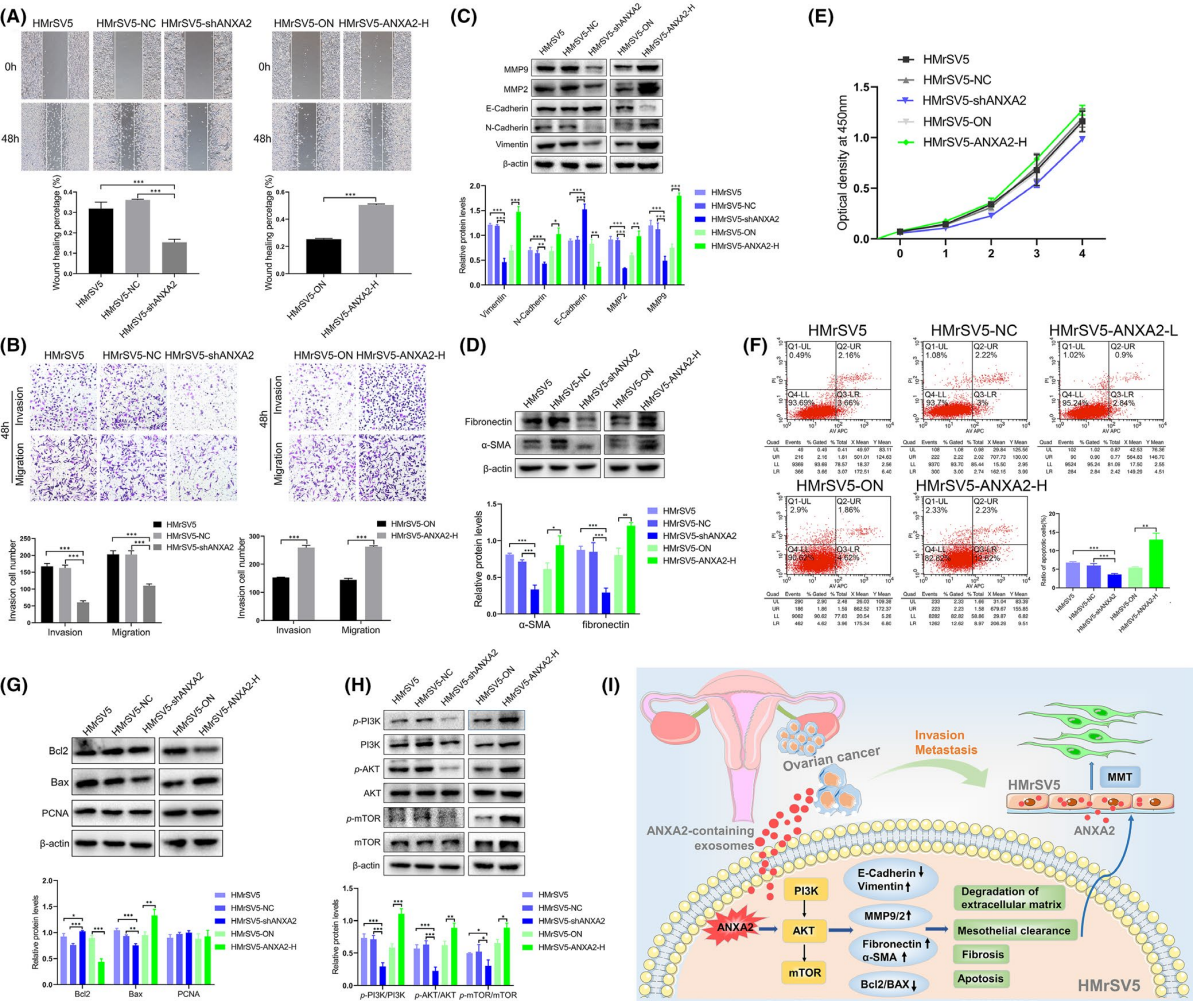
Effect of ANXA2 on the biological behaviours, mesothelial‐mesenchymal transition (MMT) and PI3K/AKT/mTOR signalling pathway of HMrSV5 cells. (A) Effect of ANXA2 on the migration ability of HMrSV5 cells detected by scratch test. (B) Effect of ANXA2 on the invasion ability of HMrSV5 cells detected by Transwell assay. (C) Effect of ANXA2 on MMT molecular markers of HMrSV5 cells detected by Western blot. D: Effect of ANXA2 on the expression of α‐SMA and fibronectin in HMrSV5 cells detected by Western blot. (E) Effect of ANXA2 on the proliferation ability of HMrSV5 cells detected by CCK8. (F) Effect of ANXA2 on the apoptotic ability of HMrSV5 cells detected by flow cytometry. Q1‐UL: necrotic cells; Q2‐UR: the late apoptotic cells; Q3‐LR: the early apoptotic cells; Q4‐LL: normal cells. (G) Effect of ANXA2 on the proliferation and apoptosis‐related protein of HMrSV5 cells detected by Western blot. (H) Effect of ANXA2 on PI3K/AKT/mTOR signalling pathways in HMrSV5 cells detected by Western blot. (I) Schematic illustration of the effect of exosomal ANXA2 derived from ovarian cancer on HMrSV5 cells during the peritoneal dissemination and metastasis of ovarian cancer

To confirm the effect and mechanism of ANXA2 on the biological behaviour of HMrSV5 cells, HMrSV5 cells with stable overexpression and inhibition of ANXA2 protein were constructed by lentiviral transfection. Western blot showed that the expression level of ANXA2 in the HMrSV5‐shANXA2 group was significantly lower than that in the HMrSV5 and HMrSV5‐NC groups (both *p *< 0.05). The expression level of ANXA2 in the HMrSV5‐ANXA2‐H group was significantly higher than that in the control group (HMrSV5‐ON) (*p *< 0.05; Figure S3). We explored the effect of ANXA2 on the migration ability of HMrSV5 cells by Scratch test. The results showed that the migration ability of HMrSV5‐shANXA2 cells was significantly lower than that of HMrSV5 and HMrSV5‐NC cells (both *p *< 0.05). The migration ability of HMrSV5‐ANXA2‐H cells was significantly higher than that of HMrSV5‐ON cells (*p *< 0.05; Figure 7A).

Transwell assay was employed to detect the effect of ANXA2 on the invasion ability of HMrSV5 cells. The results showed that the invasion ability of HMrSV5‐shANXA2 cells was significantly decreased compared with that of HMrSV5 and HMrSV5‐NC cells (both *p *< 0.05); the invasion ability of HMrSV5‐ANXA2‐H cells was significantly increased compared with that of HMrSV5‐ON cells (*p *< 0.05; Figure 7B).

We further detected the effect of ANXA2 on MMT capacity and fibrosis in HMrSV5 cells, the result showed that, compared with HMrSV5 or HMrSV5‐NC cells, HMrSV5‐shANXA2 cells showed higher expression levels of the epithelial marker protein E‐cadherin and lower expression of mesenchymal markers vimentin and N‐cadherin as well as matrix metalloproteinase (MMP)2 and MMP9 (both *p *< 0.05; Figure 7C). With overexpression of AXNA2, the expression level of E‐cadherin was downregulated, and those of vimentin, N‐cadherin, MMP2 and MMP9 were highly increased (*p* < 0.05; Figure 7C). Moreover, compared with those in the HMrSV5 and HMrSV5‐NC groups, the expression levels of α‐SMA and fibronectin in the HMrSV5‐shANXA2 group were significantly decreased (both *p *< 0.05). Compared with that in the HMrSV5‐ON group, the expression of α‐SMA and fibronectin in the HMrSV5‐ANXA2‐H group was increased (*p *< 0.05; Figure 7D).

### 3.8 Effect of ANXA2 on the proliferation, apoptosis and PI3K/AKT/mTOR signalling pathway of HMrSV5 cells

Cell Counting Kit‐8 was utilized to detect the role of ANXA2 protein on the proliferation ability of HMrSV5 cells. The results showed that, compared with HMrSV5 and HMrSV5‐NC groups, the proliferation ability of HMrSV5‐shANXA2 cells showed no significant change (*p *> 0.05; Figure 7E); compared with HMrSV5‐ON group, the proliferation ability of HMrSV5‐ANXA2‐H cells showed no significant change (*p *> 0.05; Figure 7E).

Flow cytometry showed that the apoptotic ability of HMrSV5 cells was reduced after inhibiting the expression of ANXA2 and significantly enhanced after overexpression of ANXA2 (*p *< 0.05; Figure 7F). Western blot showed that, compared with HMrSV5 and HMrSV5‐NC groups, the expression of BAX in the HMrSV5‐shANXA2 group decreased, the expression of Bcl2 increased (*p *< 0.05) and the expression of proliferating cell nuclear antigen (PCNA) did not change significantly (*p *> 0.05). Compared with HMrSV5‐ON group, the expression of BAX in HMrSV5‐ANXA2‐H cells increased, the expression of Bcl2 decreased (*p* < 0.05) and the expression of PCNA did not change significantly (*p* > 0.05; Figure 7G).

We further detected the effect of ANXA2 protein on the PI3K/AKT/mTOR signalling pathways in HMrSV5 cells with Western blot. The results showed that the expression levels of *p*‐PI3K, *p*‐AKT and *p*‐mTOR were significantly decreased in the HMrSV5‐shANXA2 group compared with those in the HMrSV5 and HMrSV5‐NC groups (both *p *< 0.05), and the expression levels of these proteins were upregulated in the HMrSV5‐ANXA2‐H group compared with those in the HMrSV5‐ON group (*p *< 0.05; Figure 7H).

## 4 DISCUSSION

In recent years, accumulating studies have shown that, as an important component of the tumour microenvironment, exosomes were involved in the occurrence, development, invasion and metastasis of various cancers through intercellular communication.^30^, ^31^, ^32^, ^33^ Moreover, tumour cell‐derived exosomes can regulate biological characteristics and signalling pathways of recipient cells, and reshape the tumour microenvironment through a delivery of multiple targeted molecules including recombinant lysyl oxidase‐like protein 4 (LOXL4), miR‐122 and miR‐222‐3p.^34^, ^35^, ^36^, ^37^ Emerging evidence suggested that exosomes could participate in malignant progression and peritoneal metastasis of ovarian cancer. For example, molecules such as CD44 and miR‐99a‐5p in exosomes derived from ovarian cancer cells can promote the MMT of HMrSV5 cells, leading to adhesion and metastasis of ovarian cancer cells.^38^, ^39^ It has been reported that ANXA2 can be transported from cell to cell by exosomes in a Ca^2+^‐dependent manner during the formation and release of exosomes.^28^ However, the function and mechanism of exosomal ANXA2 on peritoneal implantation of ovarian cancer have not been fully elucidated.

Researches have shown that exosomal ANXA2 derived from breast cancer cells can not only promote angiogenesis in a tissue plasminogen activator (tPA) ‐dependent manner but also activate multiple signalling pathways and regulate the secretion of factors in macrophages.^40^ Additionally, exosomal ANXA2 derived from serum was associated with malignant progression and angiogenesis in triple‐negative breast cancer.^41^ In this study, we firstly confirmed that ANXA2 protein in exosomes derived from ovarian cancer varies with the content of ANXA2 in the host cells, and the expression of ANXA2 was upregulated in HMrSV5 cells after co‐cultured with OVCAR3‐exo or ES‐2‐exo, indicating that ANXA2 can not only regulate the formation and release of exosomes secreted by ovarian cancer cells, but also transfer to HMrSV5 cells via exosomes, thus having potential effect on the biological characteristics of target cells.

Researches have confirmed that integrin α5β1/AEP and CD44 mediated by exosomes derived from ovarian cancer cells can induce the destruction of the mesothelial barrier.^10^, ^38^ Exosomal miR‐99a‐5p derived from ovarian cancer cells promotes tumour invasion and metastasis by regulating the expression of fibronectin and vitronectin in HMrSV5 cells,^39^ suggesting biological cargos derived from exosome could perform important effects on recipient cells. We further confirmed that exosomal ANXA2 derived from ovarian cancer can promote the migration, invasion and apoptosis of HMrSV5 cells, indicating that ovarian cancer cells can transfer their tumour characteristics to HMrSV5 cells through exosomal ANXA2, and further to form a pre‐metastasis microenvironment suitable for ovarian cancer cell adhesion and colonization. However, during this process, exosomal ANXA2 derived from ovarian cancer cells has no significant effect on the proliferation of HMrSV5 cells, which may act in the early stage of ovarian cancer intraperitoneal implantation, and the proliferation capacity is not sufficient to change, or the process was affected and regulated by other proteins and nucleic acids which warrants further investigation.

A series of studies have indicated that exosomes not only participate in modulating EMT of tumour cells but are also involved in the MMT of mesothelial cells.^42^ During abdominal implantation and metastasis of tumours, exosomes have the potential function to reshape the peritoneal microenvironment to promote adhesion and metastasis of cancers.^43^ It is reported that gastric cancer‐derived exosomes can transfer various biological molecules to induce MMT of HMrSV5 cells, such as FasL, MMP2, nicotinamide N‐methyltransferase (NNMT), tripartite motif‐containing protein 3 (TRIM3), miR‐106 and miR‐21‐5p, which could regulate biological characteristics of HMrSV5 cells, thus facilitating metastasis of gastric cancer cells.^44^, ^45^, ^46^, ^47^, ^48^ Studies have shown that PI3K/AKT/mTOR pathway played critical roles in the development of cancer,^49^ which can be abnormally activated in a variety of tumours and promote the growth, invasion, metastasis and EMT of tumours, such as ovarian cancer, breast cancer, gastric cancer, lung cancer, and head and neck squamous cell carcinoma.^50^, ^51^, ^52^, ^53^, ^54^ In this study, we successfully confirmed that, after exosomal ANXA2 derived from ovarian cancer uptaken by HMrSV5, ANXA2 protein can not only promote migration, invasion, apoptosis and MMT of HMrSV5 through downstream PI3K/AKT/mTOR pathway, but also accelerate the degradation of extracellular matrix, which provided pre‐metastatic niche for peritoneal invasion and metastasis of ovarian cancer. The above studies provide sufficient evidences that how the exosomal ANXA2 regulate biological behaviour and MMT of HMrSV5, providing new insights into the underlying mechanism of peritoneal metastasis of ovarian cancer.

## 5 CONCLUSIONS

In conclusion, to the best of our knowledge, we demonstrated for the first time that the expression of ANXA2 in exosomes derived from ovarian cancer cells varies with the content of ANXA2 in these cells. Furthermore, exosomal ANXA2 can not only regulate the migration, invasion and apoptosis abilities of HMrSV5 cells but also induce MMT, degradation of the extracellular matrix and fibrosis through the PI3K/AKT/mTOR signalling pathway, thus remodelling the pre‐metastasis microenvironment and facilitating peritoneal metastasis (Figure 7I). However, further verification by clinical samples and more animal experiments are still required in future. Taken together, our observations shed light on the function and mechanism by which exosomal ANXA2 derived from ovarian cancer cells promotes the migration, invasion and apoptosis abilities of HMrSV5 cells, providing credible evidence to clarify the mechanism of peritoneal implantation and metastasis of ovarian cancer.

## CONFLICT OF INTEREST

The authors have declared that no competing interest exists.

## AUTHOR CONTRIBUTIONS

**Lingling Gao:** Formal analysis (lead); Investigation (lead); Methodology (lead); Resources (lead); Validation (lead); Writing‐original draft (lead); Writing‐review & editing (lead). **Xin Nie:** Data curation (equal); Investigation (equal); Supervision (equal). **Rui Gou:** Investigation (equal); Validation (equal). **Yuexin Hu:** Investigation (equal); Validation (equal). **Hui Dong:** Investigation (supporting); Writing‐original draft (supporting). **Xiao Li:** Data curation (supporting); Visualization (supporting). **Bei Lin:** Funding acquisition (supporting); Project administration (supporting); Resources (supporting); Supervision (supporting).

## Supporting information

Figure S1‐S3

## Data Availability

Data available on request from the authors.
