# Supplementary material for: Exosomal ANXA2 derived from ovarian cancer cells regulates epithelial‐mesenchymal plasticity of human peritoneal mesothelial cells
Source: J Cell Mol Med. 2021 Nov 1;25(23):10916–29. doi: 10.1111/jcmm.16983 (PMC8642686; doi:10.1111/jcmm.16983)
Supplement: Supplementary file 1 — Figure S1‐S3 [file JCMM-25-10916-s001.docx]

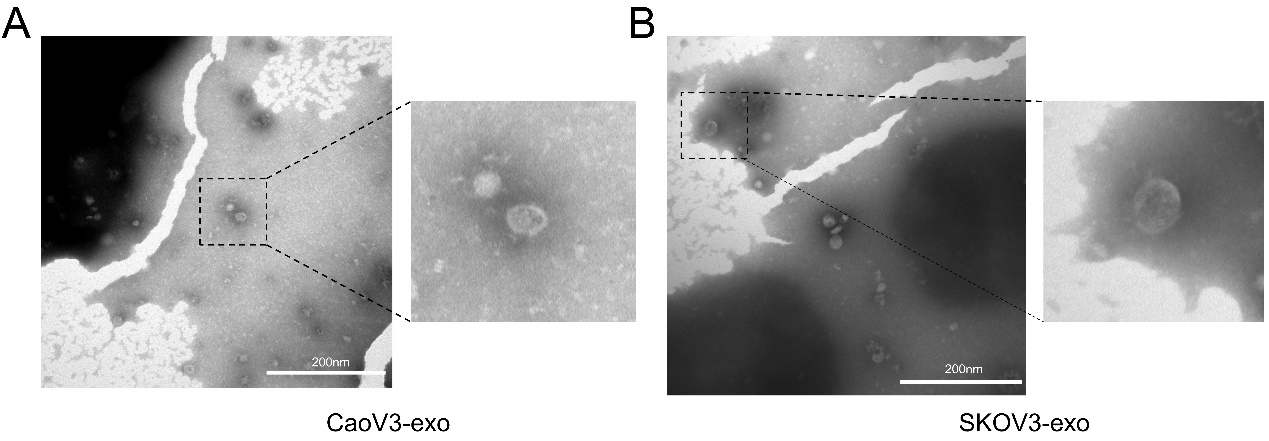


Figure S1 Typical morphology of exosomes derived from CaoV3 (A) and SKOV3 (B) cells observed by transmission electron microscope (TEM).

**
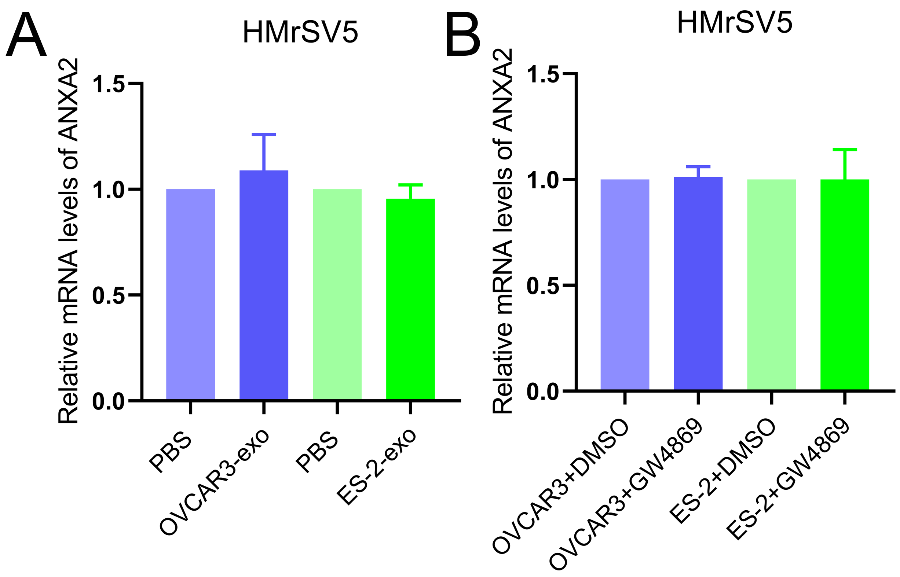
**

Figure S2 The changes of ANXA2 mRNA level in HMrSV5 cells

A: The level of ANXA2 mRNA in HMrSV5 cells co-cultured with OVCAR3-exo and ES-2-exo detected by Real-time PCR. B: The level of ANXA2 mRNA in HMrSV5 cells co-cultured with exosome inhibitor GW4869 detected by Real-time PCR


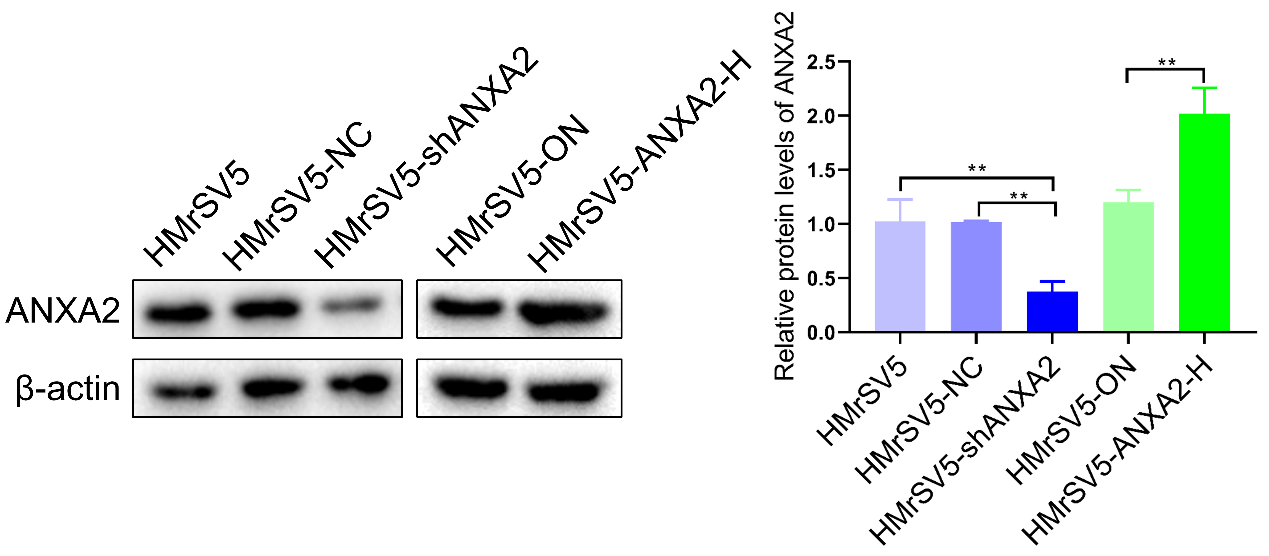


Figure S3 Protein levels of ANXA2 in HMrSV5 cells with up- and down-regulated ANXA2 detected by western blot.
